# The birth of a new journal: JHLT Open

**DOI:** 10.1016/j.jhlto.2023.100001

**Published:** 2023-08-05

**Authors:** Dirk Van Raemdonck

**Affiliations:** a Department of Thoracic Surgery, University Hospitals Leuven, Leuven, Belgium; b Department of Chronic Diseases and Metabolism, Katholieke Universiteit Leuven, Leuven, Belgium

“A new child is born” is a song by the late famous Irish singer and musician Sinéad O′Connor.^1^ Likewise, a new journal has been born in the publishing portfolio of the International Society for Heart and Lung Transplantation (ISHLT): the *Journal of Heart and Lung Transplantation – Open* (*JHLT Open*). I am honored and proud to be appointed as the first Editor-in-Chief (EIC) of our newborn.

*JHLT Open* will supplement and complement the *Journal of Heart and Lung Transplantation* (*JHLT*), ISHLT’s flagship journal,^2^ ranked as the number one journal in the transplantation category by impact factor. I am looking forward to closely working with Daniel R. Goldstein, experienced EIC of our older child. This new publication aims to extend the reach and success of ISHLT and to help shape the future of thoracic transplantation, mechanical circulatory support, and pulmonary vascular disease globally for patients suffering from end-stage heart and lung failure. Both journals provide a platform for all professionals to disseminate important novel findings that ultimately drive innovation and lead to improved patient outcomes.

*JHLT Open* is a gold open-access journal, meaning that the articles are available immediately, permanently, and universally. This peer-reviewed evidence-based journal is dedicated to publishing high-quality information that will be freely accessible and downloadable to the global advanced heart and lung disease community. Together with our publisher Elsevier, we strive to get the journal indexed in PubMed Central and to obtain an impact factor by Clarivate in the nearby future. To provide gold open access, this journal has a publication fee (Article Publishing Charge, APC) which needs to be met by the authors or their research funders for each article published open access. This ensures articles will be immediately and permanently free to access by everyone. APCs will be waived for papers published in *JHLT Open* in 2023 and discounted 50% in 2024 before they become full-priced for accepted publications in 2025.

*JHLT Open* will publish after revision or re-review good-quality full-length and review articles, case reports, and short communications that are being transferred from *JHLT*. Without relaxing rigorous standards of peer review, this article transfer model allows publication of good-quality papers that could previously not be accepted in *JHLT* because of space limitations. *JHLT Open* offers ISHLT members an extension to the publication forum for papers with diverse authorship, including many early-career professionals and researchers focusing on developments in the domains of heart and lung transplantation, mechanical circulatory support, treatment for pulmonary vascular disease, pediatric thoracic organ transplantation, and post-transplant infectious disease. In addition, practice standards and guidelines and expert consensus reports are being considered for publication that is hoped to rapidly increase the citations to *JHLT Open* papers and to contribute for obtaining an impact factor in the coming years. With the help of invited guest editors, virtual special issues can be published on specific topics as proposed by each of the 4 ISHLT interdisciplinary networks and 10 professional communities. Published papers will be grouped together online as these get accepted to create a virtual special issue digging into a topic that is of interest to an individual group of ISHLT members.

*JHLT Open* offers a unique opportunity to our clinical professionals and researchers to publish their work and make high-quality information available to readers around the globe, improving their excellent medical care for patients, and bringing comprehensive scientific knowledge to the thoracic transplant community. It is an additional vehicle to the benefit of ISHLT members. As the new Editor-in-Chief, it is my hope and wish that you will all embrace and pamper our newest family member by submitting your good scientific work. With your support, our youngest vessel hopefully one day will sail with the same scientific prestige alongside our flagship *JHLT*.

## Disclosure statement

Dirk Van Raemdonck reports a relationship with International Society for Heart and Lung Transplantation that includes: consulting or advisory.

DVR receives an editorial stipend from the International Society for Heart and Lung Transplantation for his position as Editor-in-Chief of the Journal of Heart and Lung Transplantation Open. DVR is supported by a research grant from the Broere Charitable Foundation.
